# Physico-Chemical Properties of Copper-Doped Hydroxyapatite Coatings Obtained by Vacuum Deposition Technique

**DOI:** 10.3390/ma17153681

**Published:** 2024-07-25

**Authors:** Yassine Benali, Daniela Predoi, Krzysztof Rokosz, Carmen Steluta Ciobanu, Simona Liliana Iconaru, Steinar Raaen, Catalin Constantin Negrila, Carmen Cimpeanu, Roxana Trusca, Liliana Ghegoiu, Coralia Bleotu, Ioana Cristina Marinas, Miruna Stan, Khaled Boughzala

**Affiliations:** 1 Faculty of Sciences, University de Gafsa, Route de Tozeur, Gafsa 2112, Tunisia; yassin.bn.ali27@gmail.com; 2 National Institute of Materials Physics, Atomistilor Street, No. 405A, 077125 Magurele, Romania; ciobanucs@gmail.com (C.S.C.); simonaiconaru@gmail.com (S.L.I.); catalin.negrila@infim.ro (C.C.N.); ghegoiuliliana@gmail.com (L.G.); 3 Faculty of Electronics and Computer Science, Koszalin University of Technology, Śniadeckich 2, PL 75-453 Koszalin, Poland; 4 Department of Physics, Norwegian University of Science and Technology (NTNU), Realfagbygget E3-124 Høgskoleringen 5, NO 7491 Trondheim, Norway; steinar.raaen@ntnu.no; 5 Faculty of Land Reclamation and Environmental Engineering, University of Agronomic Sciences and Veterinary Medicine of Bucharest, 59 Marasti Blvd, 011464 Bucharest, Romania; carmencimpeanu@yahoo.com; 6 National Centre for Micro and Nanomaterials, University Politehnica of Bucharest, 060042 Bucharest, Romania; truscaroxana@yahoo.com; 7 Department of Cellular and Molecular Pathology, Stefan S. Nicolau Institute of Virology, 030304 Bucharest, Romania; cbleotu@yahoo.com; 8 Research Institute of the University of Bucharest (ICUB), University of Bucharest, 060023 Bucharest, Romania; ioana.cristina.marinas@gmail.com; 9 The Academy of Romanian Scientist, 050711 Bucharest, Romania; 10 Department of Microbiology, Faculty of Biology, University of Bucharest, 1-3 Aleea Portocalelor Str., District 5, 060101 Bucharest, Romania; 11 Department of Biochemistry and Molecular Biology, Faculty of Biology, University of Bucharest, 91-95 Splaiul Independentei, 050095 Bucharest, Romania; miruna.stan@bio.unibuc.ro; 12 Higher Institute of Technological Studies of Ksar Hellal, Ksar-Hellal 5070, Tunisia; khaledboughzala@gmail.com

**Keywords:** copper-doped hydroxyapatite, coatings, vacuum deposition, biocompatibility, antimicrobial activity

## Abstract

The hydroxyapatite and copper-doped hydroxyapatite coatings (Ca_10−x_Cu_x_(PO_4_)_6_(OH)_2_; x_Cu_ = 0, 0.03; HAp and 3CuHAp) were obtained by the vacuum deposition technique. Then, both coatings were analyzed by the X-ray diffraction (XRD), scanning electron microscopy (SEM), atomic force microscopy (AFM), X-ray photoelectron spectroscopy (XPS), Fourier transform infrared spectroscopy (FTIR) and water contact angle techniques. Information regarding the in vitro antibacterial activity and biological evaluation were obtained. The XRD studies confirmed that the obtained thin films consist of a single phase associated with hydroxyapatite (HAp). The obtained 2D and 3D SEM images did not show cracks or other types of surface defects. The FTIR studies’ results proved the presence of vibrational bands characteristic of the hydroxyapatite structure in the studied coating. Moreover, information regarding the HAp and 3CuHAp surface wettability was obtained by water contact angle measurements. The biocompatibility of the HAp and 3CuHAp coatings was evaluated using the HeLa and MG63 cell lines. The cytotoxicity evaluation of the coatings was performed by assessing the cell viability through the MTT assay after incubation with the HAp and 3CuHAp coatings for 24, 48, and 72 h. The results proved that the 3CuHAp coatings exhibited good biocompatible activity for all the tested intervals. The ability of *Pseudomonas aeruginosa* 27853 ATCC (*P. aeruginosa*) cells to adhere to and develop on the surface of the HAp and 3CuHAp coatings was investigated using AFM studies. The AFM studies revealed that the 3CuHAp coatings inhibited the formation of *P. aeruginosa* biofilms. The AFM data indicated that *P. aeruginosa*’s attachment and development on the 3CuHAp coatings were significantly inhibited within the first 24 h. Both the 2D and 3D topographies showed a rapid decrease in attached bacterial cells over time, with a significant reduction observed after 72 h of exposure. Our studies suggest that 3CuHAp coatings could be suitable candidates for biomedical uses such as the development of new antimicrobial agents.

## 1. Introduction

In the last few years, significant efforts have been undertaken by the scientific community in order to enhance the biocompatibility of commonly used implant materials in orthopedics/stomatology. One potential solution purposed involves the deposition of bioactive coatings, such as hydroxyapatite (HAp), on the surface of the implants [1]. Hydroxyapatite is a bioceramic that has attracted researchers’ attention because of its structural and chemical resemblance to the main inorganic component of bone tissue. Hydroxyapatite has been used as a coating material for metallic implants due to its excellent cytocompatibility, ability to stimulate cellular functions, and good osteoconductivity [2]. Nonetheless, previous studies have shown that natural hydroxyapatite, which is found in the mineral phases of bone, dentin, and enamel, possesses a chemical composition that is different from synthetic HAp [1,2,3]. Typically, natural HAp encompasses a wide range of trace elements, such as silicon (Si), fluorine (F), magnesium (Mg), strontium (Sr), zinc (Zn), etc., each possessing distinct biological features [4]. The appearance of post-surgical infections can prevent proper bone integration and cause tissue necrosis, posing serious health risks to patients and altering their quality of life [5,6,7]. The increase in antibiotic-resistant bacteria is driving research toward new solutions, including the use of metallic ions for their antibacterial properties [5,8]. It is well known that copper (Cu) is a crucial trace element for all organisms and that it is vital for a wide range of physiological functions, including energy production, respiration and tissue development [9,10]. On the other hand, it serves as an enzyme cofactor in many metabolic processes. Furthermore, copper is crucial for bone mineralization and for osteoblast activity [9,10,11,12,13,14,15]. Insufficient levels of copper are associated with a range of medical conditions, such as myeloneuropathy, leucopenia, anemia, and Menke’s disease [10,13]. Moreover, high levels of Cu are harmful for the human body and lead to Wilson’s disease [10,14]. Moreover, copper possesses excellent antimicrobial activity, having been used from ancient times by the Egyptians for maintaining the purity of water [10,15]. Therefore, nowadays, the copper ion is regarded as a promising doping agent due to its pronounced antibacterial activity and low cytotoxicity.

According to the study conducted by Hidalgo-Robatto and coworkers [16], infections related to prosthetic implants and medical devices are mainly caused by certain bacteria, including *Staphylococcus epidermidis* and *Staphylococcus aureus* (Gram-positive), as well as *Escherichia coli* and *Pseudomonas aeruginosa* (Gram-negative). Therefore, the enriching of biomaterials that cover implants/medical devices with antimicrobial agents could represent a promising alternative as a systemic treatment with antibiotics [16]. For example, *Pseudomonas aeruginosa* (*P. aeruginosa*), a Gram-negative bacterium, is known for its multidrug-resistant and extensively drug-resistant strains, which frequently cause severe infections [17]. This fact presents significant challenges in selecting effective antimicrobial treatments due to their resistance [17]. The study reported by Jacobs et al. [5], which was conducted on the copper-doped biphasic calcium phosphate materials, underlined their good antibacterial activity against Gram-positive (methicillin-resistant *Staphylococcus aureus* and methicillin-sensitive) and Gram-negative (*Pseudomonas aeruginosa* and *Escherichia coli*) strains [5]. Other similar studies that have previously been reported revealed that the antibacterial efficiency of copper-doped biomaterials against *Pseudomonas aeruginosa* is dose- and incubation time-dependent [10]. Information about copper-doped hydroxyapatite was provided in various studies conducted by Bazin et al. [18] and Noori et al. [19]. Thus, the in vitro biological studies conducted by Bazin et al. [18] on the MC3T3-E1 cell line revealed that the incorporation of copper into the sintered hydroxyapatite ceramics did not alter the proliferation and cell adhesion, behavior that confirms the sample’s biocompatibility [18]. Furthermore, the results of the studies performed by Noori et al. [19] underlined that copper-doped hydroxyapatite does not disrupt the differentiation of stem cells into osteoblasts, which is a crucial process for the regeneration of bone tissue [19]. Also, the biocompatibility of copper-doped hydroxyapatite is influenced by the copper content [18,19,20]. Previous studies [20] showed that concentrations of copper up to 5% typically promote cellular growth and functionality, whereas higher levels of copper can lead to toxic effects on cells [20]. The groundbreaking aspect of this work lies in the development of new antimicrobial and biocompatible coatings using 3CuHAp powders through a cost-efficient method: vacuum deposition. Moreover, another aspect underlining the novelty and originality of this work is the achievement (for the first time) of continuous and pure 3CuHAp layers through vacuum deposition.

This study aimed at the development for the first time of copper-doped hydroxyapatite coatings (Ca_10−x_Cu_x_(PO_4_)_6_(OH)_2_; x_Cu_ = 0.03; 3CuHAp) by vacuum deposition. Also, this study explored the physico-chemical properties of 3CuHAp coatings, together with their toxic effects on cells and their antibacterial activity. Techniques such as X-ray diffraction (XRD), X-ray photoelectron spectroscopy (XPS), atomic force microscopy (AFM), scanning electron microscopy (SEM), Fourier transform infrared spectroscopy (FTIR) and water contact angle were employed in order to obtain valuable information about the features of the HAp and 3CuHAp coatings. In summary, this work pioneers a novel approach by combining superior physico-chemical and biological properties and cost efficiency in the development of coatings based on HAp and 3CuHAp. These coatings have the potential to improve medical devices/implants, enhancing overall health.

## 2. Materials and Methods

### 2.1. Materials

#### 2.1.1. Synthesis of Hydroxyapatite (HAp) and Copper-Doped Hydroxyapatite (3CuHAp)

For the synthesis of undoped hydroxyapatite coatings (Ca_10_Cu(PO_4_)_6_(OH)_2_; Ca/P ratio equal with 1.67; referred to as HAp), the steps described in our previous work [21] were followed. Firstly, two solutions were obtained, containing 0.1 mol of Ca(NO_3_)_2_∙4H_2_O (Sigma Aldrich, St. Louis, MO, USA) and 0.06 mol of (NH_4_)_2_HPO_4_ (Alfa Aesar, Karlsruhe, Germany), respectively. Then, the solution containing Ca(NO_3_)_2_∙4H_2_O was slowly added into the (NH_4_)_2_HPO_4_ solution. During the synthesis, the pH value was maintained at 11. The resulting mixture was stirred at 100 °C for 4 h, then centrifuged and washed for 5 times. Finally, the HAp precipitate was dried at 100 °C and used for the vacuum deposition of the HAp coatings.

A similar procedure was used for obtaining of the copper-doped hydroxyapatite powders (with the chemical formula: Ca_10−x_Cu_x_(PO_4_)_6_(OH)_2_; where x_Cu_ = 0.03; referred to as 3CuHAp). During the synthesis, the [Ca + Cu]/P ratio was maintained at 1.67. Briefly, the Cu(NO_3_)_2_·3H_2_O (Alfa Aesar, Karlsruhe, Germany) was dissolved with the Ca(NO_3_)_2_∙4H_2_O. The next steps were carried out identically to the those described for the hydroxyapatite powders’ synthesis. Finally, the 3CuHAp precipitate was dried at 100 °C and used for the vacuum deposition of the 3CuHAp coatings.

#### 2.1.2. Development of Hydroxyapatite (HAp) and Copper-Doped Hydroxyapatite (3CuHAp) Coatings

**Figure 1:**
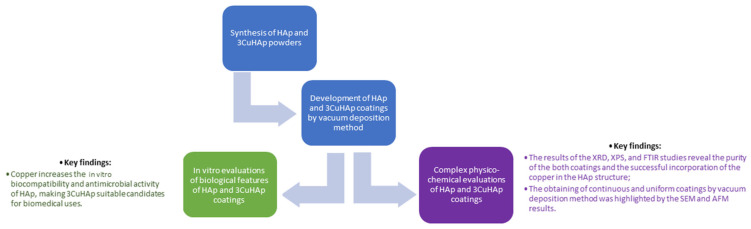
Schematic representation of the synthesis, characterization techniques and key findings.

The deposition of the HAp and 3CuHAp coatings was performed on silicon (Si) substrates. Prior to the vacuum deposition process, the substrates underwent multiple cleanings with acetone and were air-dried at 40 °C. The deposition parameters for the coatings were consistent with the ones presented by Predoi et al. [22]. Briefly, the deposition process took place in a high vacuum environment (~10^−6^ mbar). The powders were thermally evaporated from a W boat, slowly increasing the current through them until the melting state became visible. Afterward, the melt was completely evaporated in about 120 s. Finally, the HAp and 3CuHAp coatings were thermally treated at 500 °C in air. Figure 1 presents a schematic representation of the synthesis, characterization techniques and key findings of the studies conducted on the HAp and 3CuHAp coatings. Furthermore, the vacuum deposition technique used for the development of the 3CuHAp coatings is a versatile and cost-efficient method. While the setup cost is moderate, the ongoing operation and maintenance costs are manageable. Compared to other deposition techniques, vacuum deposition is cost-effective due to its simplicity and high deposition rate. Other deposition techniques use expensive equipment that requires considerable operation and maintenance costs.

### 2.2. Characterizations of 3CuHAp Coatings

#### 2.2.1. X-ray Diffraction (XRD)

The X-ray diffraction (XRD) studies were conducted using a Bruker D8 Advance X-ray diffractometer (manufactured by Bruker in Karlsruhe, Germany). The XRD experimental data were acquired within the 20° to 60° (2θ) range, with a step size of 0.02°, using Cu Kα radiation (wavelength λ = 1.5418 Å).

#### 2.2.2. Scanning Electron Microscopy (SEM)

The FEI Quanta Inspect F scanning electron microscope (manufactured by FEI Company, Hillsboro, OR, USA) was used in order to evaluate the surface morphology of both coatings. Additionally, the microscope is equipped with an energy-dispersive X-ray (EDS) attachment, which enabled the evaluation of the chemical composition of the HAp and 3CuHAp coatings. More than that, the thickness of both coatings was evaluated using SEM transversal cross-section images. The 3D SEM images were obtained with the aid of ImageJ 1.51j8 software [23].

#### 2.2.3. Atomic Force Microscopy (AFM)

For this study, an atomic force microscope (AFM, NT-MDT NTEGRA Probe NanoLaboratory system, Moscow, Russia) operated in non-contact mode was used to obtain information about the surface features of the HAp and 3CuHAp coatings [24]. The AFM studies were performed using a silicon NT-MDT NSG01 cantilever. The AFM images were recorded on a surface area of 3 × 3 µm^2^ and Gwyddion 2.55 software was used for their analysis [25].

#### 2.2.4. X-ray Photoelectron Spectroscopy (XPS)

A multimethod SPECS surface analysis system (SPECS GmbH, Berlin, Germany) operating with Al Kα monochromatic radiation (1486.6 eV) was used in order to perform the X-ray photoelectron spectroscopy (XPS) studies. The experimental conditions were reported in previous research conducted by Iconaru et al. [26]. The XPS data were processed using Spectral Data Processor v. 2.3 (SDP) software. The binding energy of the calibration using C1s was 284.8 eV.

#### 2.2.5. Fourier Transform Infrared Spectroscopy (FTIR)

The PerkinElmer Spectrum 100 FT-IR spectrometer (Waltham, MA, USA) is an instrument widely used for establishing the presence of functional groups in the structure of coatings. Experimental data were collected in the spectral range of 450–1200 cm^−1^. Additionally, following the procedure detailed in [27], the second derivative spectra of the HAp and 3CuHAp coatings were obtained. The FTIR data were analyzed using the OriginPro 2021b software (OriginLab Corporation, Northampton, MA, USA).

#### 2.2.6. Water Contact Angle Studies

Water contact angle studies were conducted under ambient conditions with the aid of a contact angle goniometer (DSA30 Kruess GmbH, Hamburg, Germany). The sessile drop method was used for the experiments. The HAp and 3CuHAp coatings underwent three repetitions of the contact angle measurement. The mean contact angle values are reported, along with the standard deviation (SD).

#### 2.2.7. In Vitro Antibacterial Activity

The antibacterial properties of the HAp and 3CuHAp coatings were evaluated against the standard *Pseudomonas aeruginosa* 27853 ATCC bacterial strain. The in vitro antibacterial assays were conducted following the protocol outlined in [28]. The antibacterial activity of the 3CuHAp coatings was assessed after 24, 48, and 72 h of incubation with the *P. aeruginosa* 27853 ATCC bacterial suspensions. The quantitative measurements of the bacterial cell survival were recorded at these intervals. For this purpose, *P. aeruginosa* suspensions with a bacterial density of approximately 5 × 10^6^ colony-forming units (CFUs)/mL, derived from 18–24 h cultures, were used in the study. The 3CuHAp coatings were incubated at 37 °C for 24, 48, and 72 h with these bacterial suspensions, and the bacterial growth was monitored over time. For each incubation period, the bacterial suspensions were collected and incubated on LB agar medium. A free bacterial suspension served as a positive control (C+). The CFU count per milliliter (CFU/mL) was determined and graphically represented as the log CFU/mL over time. The antibacterial experiments were conducted in triplicate, and the results are expressed as the mean ± standard deviation (SD). The qualitative evaluation of the bacterial cell adherence and proliferation on the surface of the 3CuHAp coatings was also studied using the atomic force microscopy (AFM) technique. For these assays, *P. aeruginosa* bacterial cells were cultured on the surface of the 3CuHAp coatings for three different time intervals. After each incubation period, the 3CuHAp coatings were removed from the culture medium, washed using sterile saline solution to remove the unattached bacterial cells, fixed with cold methanol, and prepared for visualization.

#### 2.2.8. In Vitro Biological Evaluation

The cytotoxicity of the HAp and 3CuHAp coatings was assessed using both HeLa (ATCC CRM-CCL2) and osteosarcoma MG63 (ATCC CRL-1427) cells, following a similar methodology to that described by Iconaru et al. [29]. The cells were cultured in Dulbecco’s modified Eagle’s medium (DMEM) enriched with heat-inactivated fetal bovine serum at 37 °C in an atmosphere containing 5% CO_2_. The HeLa and MG63 cells were seeded at a concentration of 1 × 10^5^ cells/well in complete medium and incubated with the HAp and 3CuHAp coatings for 24, 48, and 72 h. The cytotoxicity was evaluated by measuring the cell viability using the MTT [3-(4,5-dimethylthiazolyl)-2,5-diphenyltetrazolium bromide] reduction assay. After the incubation periods, the cells were washed with phosphate-buffered saline (PBS) and incubated with 1 mg/mL MTT solution for 2.5 h. The cell viability was determined by measuring the optical density of the medium at 595 nm using a TECAN spectrophotometer. The percentage of viable HeLa and MG63 cells was calculated relative to a control sample, which was set to 100% viability. The HeLa and MG63 cells were seeded at a concentration of 1 × 10^5^ cells/well in complete medium and maintained at 37 °C for 72 h. The morphology of the cells maintained together with the HAp and 3CuHAp coatings was observed under visible light using an Axio Observer Inverted microscope (Carl Zeiss, GMBH, Munich, Germany). In addition, the adhesion of the cells on the material was highlighted by fixing them with 70% ethanol, staining them with propidium iodide and photographing them with an Axio Observer D Inverted microscope equipped with a fluorescence module (Carl Zeiss, GMBH, Munich, Germany).

Furthermore, the cells were also visualized with the aid of an inversed trinocular metallographic microscope OX.2153-PLM (Euromex, Arnhem, The Netherlands). The metallographic microscope was equipped with an CMEX digital camera and the images were acquired with ImageFocusAlpha software (v 1.3.7.19728, Euromex, Arnhem, The Netherlands) using the 10× magnification objective. These studies were performed under ambient conditions.

## 3. Results and Discussion

**Figure 2:**
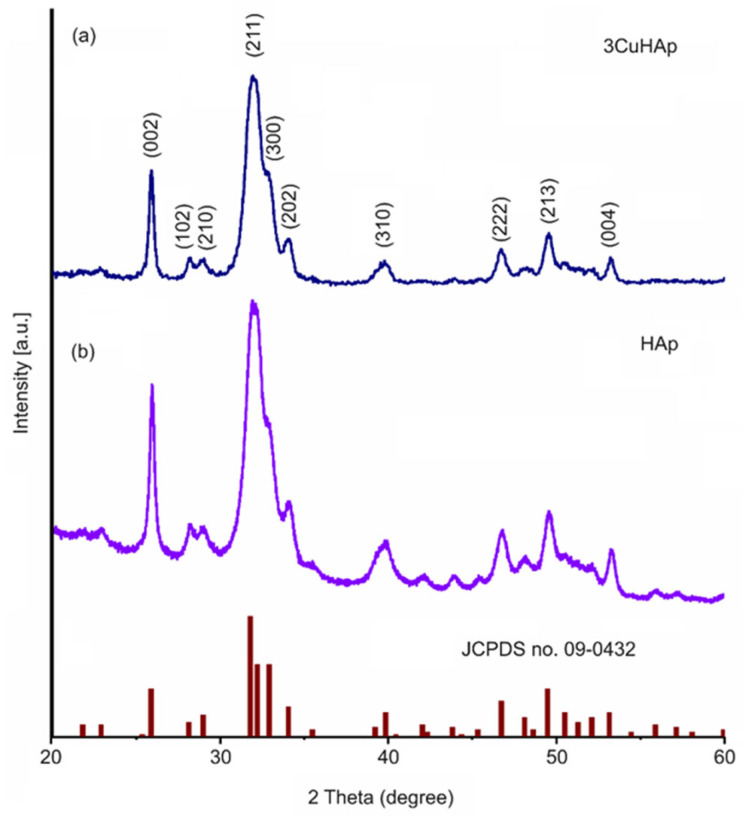
XRD pattern of the HAp (**b**) and 3CuHAp (**a**) coatings and JCPDS no. 09-0432.

The samples consisting of the HAp and 3CuHAp coatings obtained by the vacuum deposition process were analyzed from a structural point of view by X-ray diffraction (Figure 2).

The X-ray diffraction analysis of the deposited HAp and 3CuHAp coatings revealed the presence of hydroxyapatite (Figure 2a,b). This was highlighted by the diffraction pattern, which exhibited maxima associated with pure hexagonal hydroxyapatite (JCPDS no. 09-0432).

The diffraction peaks corresponded to specific crystallographic planes of the hexagonal HAp structure. These planes were (002), (210), (211), (300), (202), (310), (222), (213), (004), and (322). There were no peaks associated with impurities, indicating that the coatings were composed of a single phase of hydroxyapatite. The calculated lattice parameters were a = 9.393 Å and b = 6.869 Å for the 3CuHAp sample, while for the HAp coatings, the values were a = 9.416 Å and b = 6.881 Å. The values of the lattice parameters were lower compared to the values of pure hydroxyapatite (a = 9.418 Å and b = 6.884 Å). The cell volume in the case of the 3CuHAp sample was equal of 524.72 Å^3^, while the cell volume of the HAp coatings was 528.32 Å^3^. The cell volume of pure hexagonal HAp is 528.80 Å^3^. For the HAp (x_Cu_ = 0.00) and 3CuHAp (x_Cu_ = 0.03) samples, the crystallite size was determined using Scherrer’s equation [30,31,32]. The calculated crystallite size for the HAp sample was 19.45 nm, while for the 3CuHAp sample, it was 12.18 nm. The substitution of calcium (Ca) for copper (Cu) at the Ca (II) sites in the hydroxyapatite (HAp) lattice leads to a decrease in the lattice parameters and a contraction of the cell volume. This behavior aligns with findings from prior studies reported by Mariappan et al. [33].

**Figure 3:**
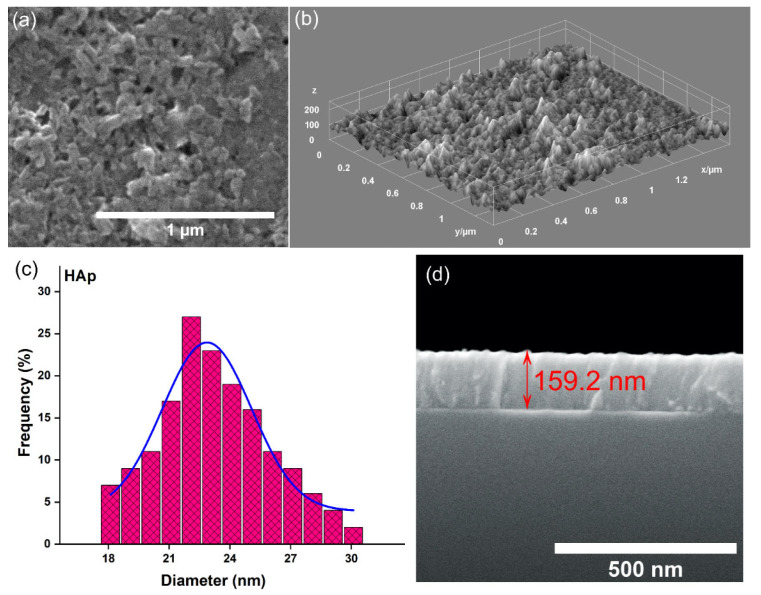
(**a**) SEM image (2D) of the HAp coatings; (**b**) SEM image (3D) of the HAp coatings; (**c**) SEM particle size distribution of the HAp coatings; and (**d**) SEM transversal cross-section image of the HAp coatings.

Figure 3 shows the 2D and 3D SEM micrographs, together with the SEM particle size distribution histogram and SEM transversal cross-section image, obtained for HAp. The SEM images (both the 2D and 3D representations) reveal the presence of a continuous coating with no evidence of cracks. Furthermore, the mean particle size obtained by SEM for HAp was 22.1 nm. The transversal cross-section image obtained for the HAp coatings deposited by vacuum deposition on the Si substrate shows that their thickness was around 159.2 nm.

**Figure 4:**
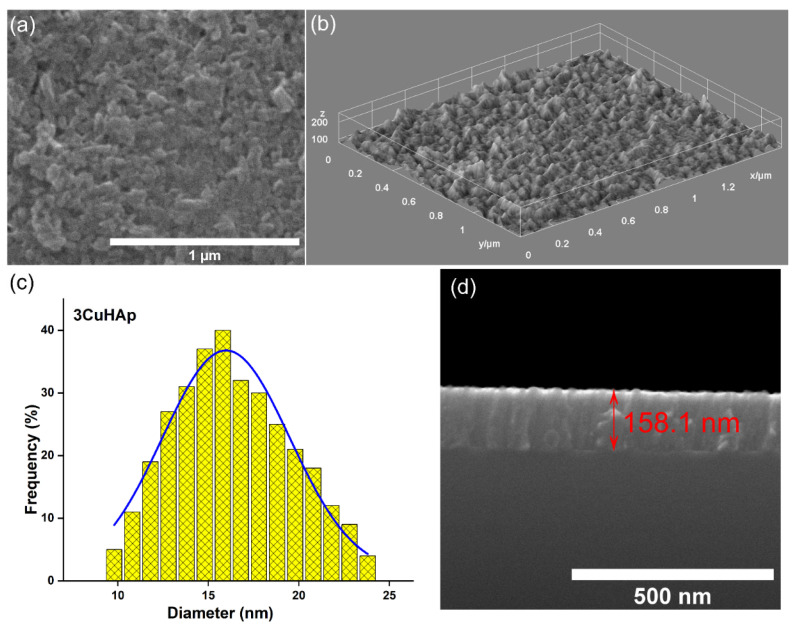
(**a**) SEM image (2D) of the 3CuHAp coatings; (**b**) SEM image (3D) of the 3CuHAp coatings; (**c**) SEM particle size distribution of the 3CuHAp coatings; and (**d**) SEM transversal cross-section image of the 3CuHAp coatings.

Both the 2D and 3D scanning electron microscope images acquired from the 3CuHAp coatings are presented (Figure 4a,b). Notably, the 3CuHAp coatings’ surface exhibits an absence of cracks and fissures. Additionally, the SEM images highlight the continuous and homogeneous surface morphology of the 3CuHAp coatings. Importantly, no other surface defects are noticeable in the obtained 2D and 3D SEM images (Figure 4a,b). The mean particle size determined via SEM was 15.8 nm for the 3CuHAp. Moreover, the SEM transversal cross-section image suggests that the thickness of the 3CuHAp coatings was around 158.1 nm.

**Figure 5:**
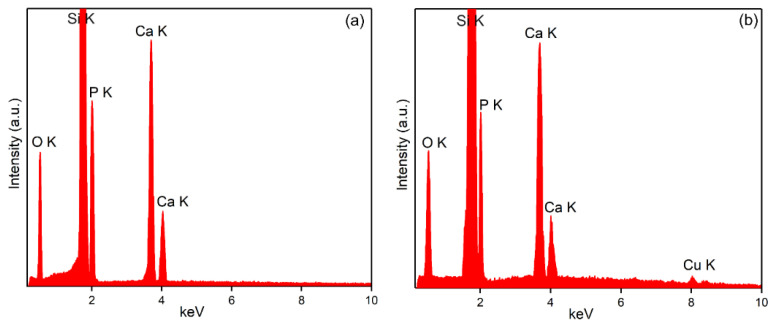
The EDS spectra obtained for the HAp (**a**) and 3CuHAp (**b**) coatings.

The EDS spectra obtained for the HAp and 3CuHAp coatings deposited on a silicon (Si) substrate by the vacuum deposition coating process are presented in Figure 5a,b. The results indicate that both coatings are pure. This conclusion is based on the absence of maxima in the EDS spectra that could be attributed to contaminants. In the EDS spectra specific to the HAp coatings (Figure 5b), only the lines associated with the next chemical elements that belong to the HAp structure are present: calcium (Ca), oxygen (O) and phosphorus (P). The EDS spectra of the 3CuHAp coatings revealed the presence of calcium (Ca), oxygen (O), phosphorus (P), and copper (Cu) chemicals. These elements collectively contribute to the composition of the Ca_10-x_Cu_x_(PO_4_)_6_(OH)_2_ structure. Additionally, both EDS spectra showed an Si line. This Si signal originates from the silicon substrate on which the HAp and 3CuHAp coatings were deposited.

**Figure 6:**
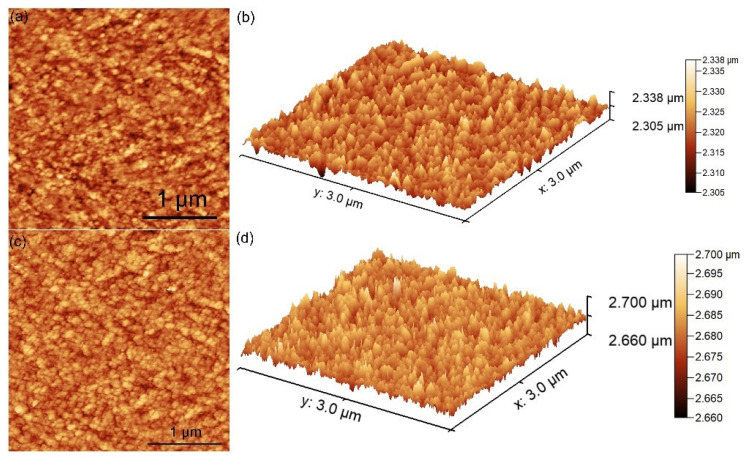
(**a**) The 2D and (**b**) 3D AFM images obtained for the HAp coatings; and (**c**) the 2D and (**d**) 3D AFM images obtained for the 3CuHAp coatings.

Atomic force microscopy (AFM) was used to examine the surface topography of the HAp and 3CuHAp coatings. The findings from the AFM studies (2D, together with their 3D representations) are illustrated in Figure 6a–d. The results of the AFM studies obtained for the hydroxyapatite coatings are presented in Figure 6a,b and indicate the presence of a uniform coating consisting of nanoagregates that are well distributed on the surface. The AFM images (2D and their 3D representations) reveal that no important cracks or fissures could be noticed on the HAp coatings’ surface. The value obtained for the roughness parameter (R_RMS_) by AFM for the HAp coatings was 19.02 nm.

Both the 2D and 3D AFM micrographs reveal the surface topography of the 3CuHAp coatings (Figure 6c,d). The AFM analysis confirms that the studied coating displays a uniform and continuous morphology. Additionally, the 2D surface images clearly show an absence of cracks or fissures, indicating that the nanoaggregates are evenly distributed across the surface of the 3CuHAp coatings. Furthermore, the value obtained from the AFM studies for the roughness parameter (R_RMS_) was equal to 18.73 nm. As can be seen, the value of R_RMS_ for the 3CuHAp coatings is smaller compared with the value of R_RMS_ obtained for the HAp coatings. This behavior indicates a decrease in roughness in the case of 3CuHAp and could be attributed to the HAp lattice distortion induced by the copper substitution.

Supplementary information about the roughness of the HAp and 3CuHAp coatings was obtained from the AFM images. The values of the roughness parameters (R_a_ and R_q_), were obtained from the AFM images and are presented in Table 1. The value obtained for the roughness average (R_a_) parameter was 15.43 nm for HAp and 15.16 nm for 3CuHAp. On the other hand, the values obtained for the root mean square (RMS) roughness (R_q_) were nearly equal, with 19.02 nm for HAp and 18.73 nm for 3CuHAp.

Table 2 depicts the values obtained for the roughness parameters (R_a_ and R_q_), as quantified from the SEM images. For the determination of the roughness parameters from the SEM images, the images were first preprocessed and the Z height was correlated from the AFM images. The results obtained from the SEM images concerning the roughness parameters of the HAp and 3CuHAp coatings are similar and could be correlated with the results determined from the AFM surface topographies.

For the HAp coatings, the value obtained for the roughness average (R_a_) parameter was 15.45 nm. Meanwhile for the 3CuHAp, the roughness average (R_a_) parameter was 14.78 nm. The root mean square (RMS) roughness (R_q_) values were 19.87 nm for HAp and 18.79 nm for 3CuHAp.

The values obtained for the R_a_ and R_q_ parameters (from AFM and SEM images) indicate that the studied samples exhibit a low roughness. Moreover, in the case of the 3CuHAp sample, a slight decrease in the roughness parameters could be noticed, a fact that could be attributed to the presence of copper ions in the HAp structure. Furthermore, according to the study reported by Osman et al. [34], a surface with a lower roughness could promote superior biological properties.

**Figure 7:**
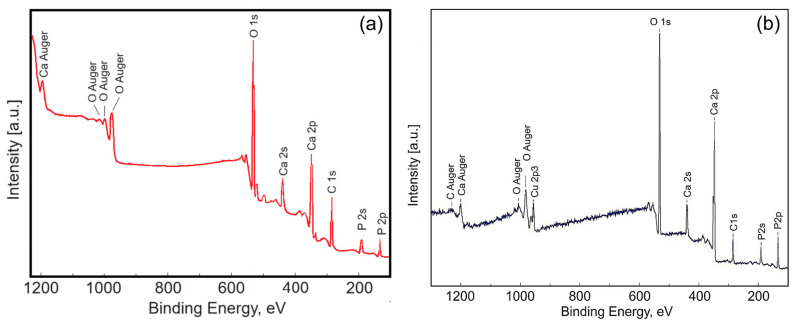
XPS survey scan of the HAp (**a**) and 3CuHAp (**b**) coatings obtained by the vacuum deposition process.

To characterize the surface of the HAp and 3CuHAp coatings, X-ray photoelectron spectroscopy was used. Figure 7 presents the XPS survey spectra of the of the HAp (x_Cu_ = 0) coatings. The qualitative analysis revealed the presence of key constituents of hydroxyapatite (Figure 7a), including calcium (Ca), phosphorus (P) and oxygen (O). The presence of copper (Cu) was observed in the general spectrum of 3CuHAp (Figure 7b). The results are in agreement with previous studies [35]. These results align with those achieved from the energy-dispersive X-ray spectroscopy (EDS) analysis, attesting to the presence of copper in the examined samples.

**Figure 8:**
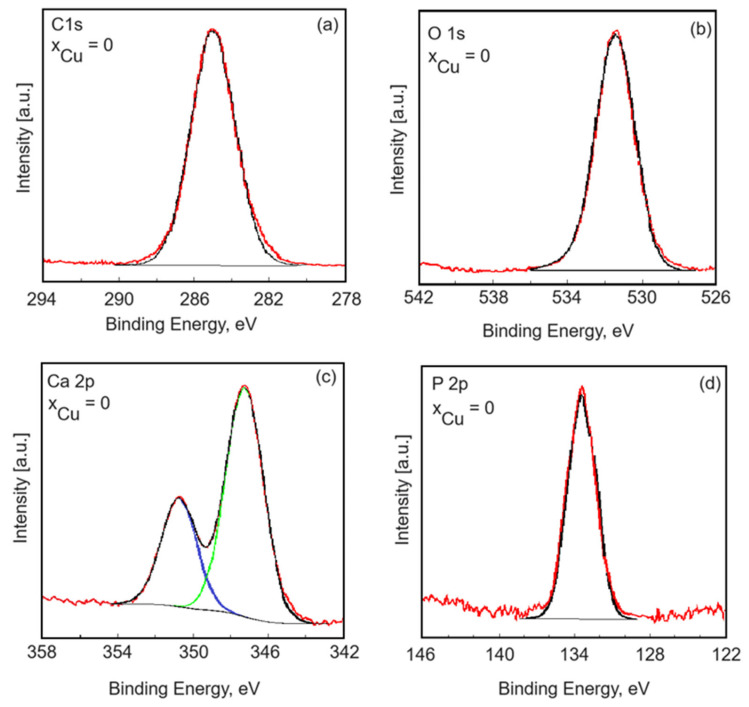
High-resolution XPS spectra of C1s (**a**), O 1s (**b**), Ca 2p (**c**) and P 2p (**d**) of the HAp coatings obtained by the vacuum deposition process.

High-energy resolution analysis of the individual peaks was performed. Thus, the high-resolution spectra of HAp for carbon, oxygen, calcium and phosphorus are presented in Figure 8. The measured binding energy (EB) scale was referenced to a C 1s at the EB value of 284.8 eV [36]. Figure 8 reveals the high-resolution C 1s spectra for the pure HAp (x_Cu_ = 0) coatings. As can be seen, we have only one component that was highlighted at 284.8 eV. This peak is associated with residual or accidental carbon. Serra et al. [37] associated the 284.8 eV peak with C–C and C–H bonds. The high-resolution XPS spectrum of O 1s for the HAp coatings shows one peak located at 531.4 eV (Figure 8b). The peak at 531.4 eV is associated with the hydroxyl groups resulting from the chemisorption of water or oxygen. The results are in line with previous studies by Moulder et al. [38]. The Ca 2p high-resolution spectrum of the HAp coatings exhibited a well-resolved doublet with two components assigned to Ca 2p3/2 and Ca 2p1/2 (Figure 8c). The peak located at about 347.2 eV shows that the calcium atoms are bound to a phosphate group (PO_4_^3−^). Following the processing of the deconvolution data, the P 2p photoelectron line consists of one single peak at a binding energy of 133.1 eV. In accordance with past XPS studies [39], the binding energy of the photoelectron peaks for Ca and P is characteristic of their full oxidation states (Ca^2+^ and P^5+^) for hydroxyapatite.

**Figure 9:**
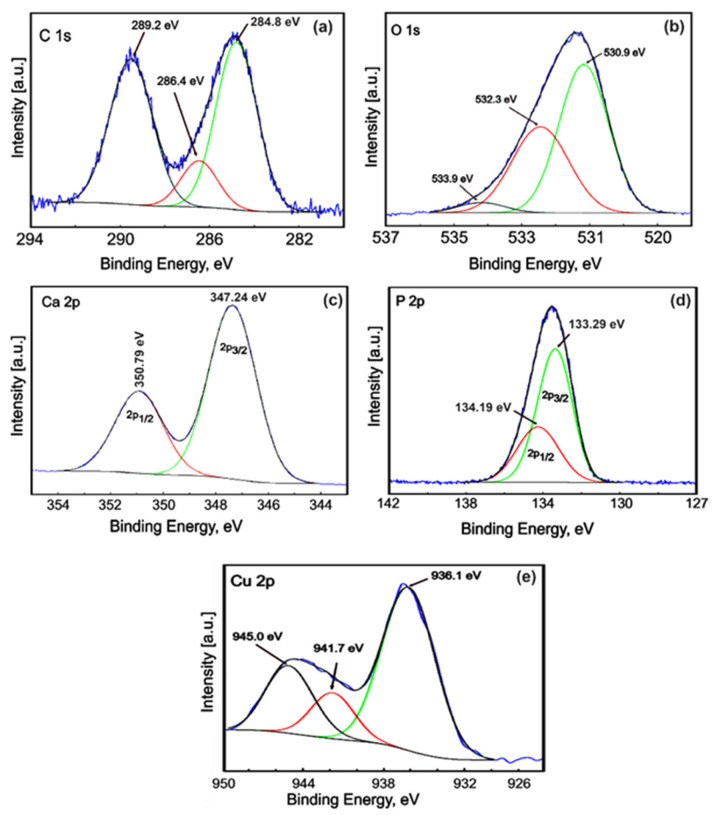
High-resolution XPS spectra of C1s (**a**), O 1s (**b**), Ca 2p (**c**), P 2p (**d**) and Cu 2p (**e**) of the 3CuHAp coatings obtained by the vacuum deposition process.

The high-resolution spectra of the 3CuHAp sample are presented in Figure 9. The high-resolution XPS spectrum of C 1s of the 3CuHAp sample shows three peaks identified at binding energies of 284.8, 286.4 and 289.2 eV. The peak at 284.8 eV is attributed to C–C and C–H single bonds [40,41]. The peaks at 286.4 eV and 289.2 eV are assigned to C–O–C bonds and O–C=O bonds [40,41]. The high-resolution XPS spectrum of O 1s for the 3CuHAp thin film shows three peaks located at 530.9 eV, 532.3 eV and 533.9 eV (Figure 9b). The peak observed at 530.9 eV is attributed to HAp (the lattice oxygen of the P O species). The peak identified at 532.3eV is associated with oxygen adsorbed on the HAp surface [42,43]. The peak located at the binding energy of 533.9 eV could be due to possible traces of water. The high-resolution spectrum for calcium (Figure 9c) shows two peaks, Ca 2p 3/2 and Ca 2p ½, which are identified at 347.24 eV and 350.79 eV, being associated with the tetravalent state (Ca^2+^), in agreement with previous studies [42]. The high-resolution spectrum of P2p for the 3CuHAp thin film is presented in Figure 9d. The P 2p 3/2 and P 2p 1/2 peaks were located at 133.20 eV and 134.19 eV, respectively. The given information states that the ratio of the areas is 2:1, and the separation occurs at an energy of 0.9 eV. The bond-binding energy is specific to PO_4_ in hydroxyapatite. In Figure 9e, the high-resolution XPS spectra of copper (Cu) reveal three distinct maxima resulting from deconvolution. These peaks occur at specific binding energies of 936.16 eV, 941.79 eV and 945.05 eV. The peak identified at 936.16 eV corresponds to the Cu2p3/2 state. The peaks noticed at 941.79 eV and 945.05 eV are shake-up peaks, suggesting the presence of Cu^2+^ species [44,45,46]. The peaks associated with Cu^1+^ [47] and CuO at binding energies of 952.3 eV and 933.3 eV [48] were not identified.

**Figure 10:**
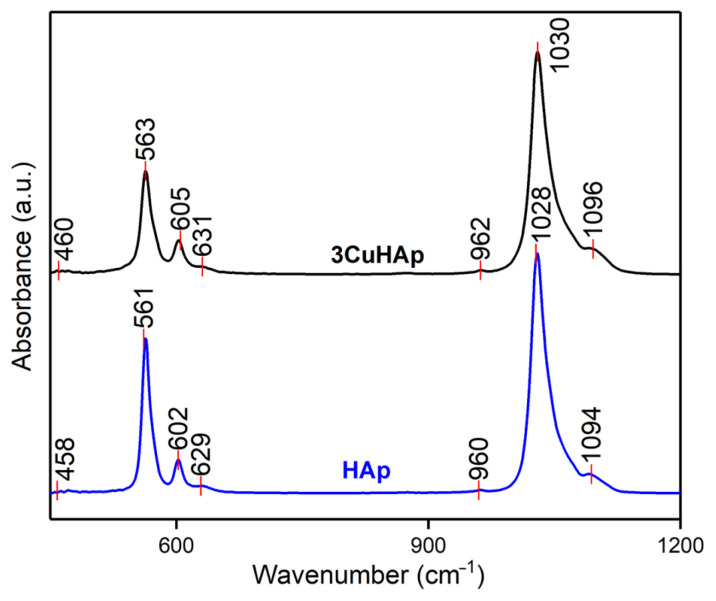
The FTIR spectra of the HAp and 3CuHAp coatings.

Figure 10 presents the absorption FTIR spectra obtained for the HAp and 3CuHAp coatings. The FTIR spectra of the HAp coatings reveal the presence of the main characteristic vibrational bands of the phosphate and hydroxyl groups from the hydroxyapatite structure. Due to the similarity, in the following, only the maxima noticed in the FTIR spectra of 3CuHAp will be discussed.

The FTIR spectra are dominated by two intense vibrational bands that are centered at ~563 cm^−1^ (ν_4_) and ~1030 cm^−1^ (ν_3_) and are characteristics of phosphate groups from HAp. Other vibrational bands that are attributed to phosphate groups’ vibrations are observed at ~460 cm^−1^ (ν_2_), ~605 cm^−1^ (ν_4_), ~962 cm^−1^ (ν_1_) and ~1096 cm^−1^ (ν_3_) [16,27,49,50,51,52,53,54,55]. The maxima observed at ~962 cm^−1^ (ν_1_) clearly indicate the presence of HAp in the 3CuHAp coatings [16,27,49,50,51,52,53,54,55]. On the other hand, the vibration peak specific to OH^−^ groups from HAp appears at ~631 cm^−1^. Furthermore, in the FTIR spectra obtained for the undoped HAp coatings presented in Figure 10, the presence of maxima could be observed that are associated with the same vibrational groups as the one observed for the 3CuHAp coatings. Also, the doping of HAp with copper led to a decrease in the FTIR maxima, together with a slight displacement of their position. No other significant vibrational bands are noticed in the FTIR spectra, a fact that indicates the coatings’ purity. Therefore, the FTIR results are in concordance with the XRD results.

**Figure 11:**
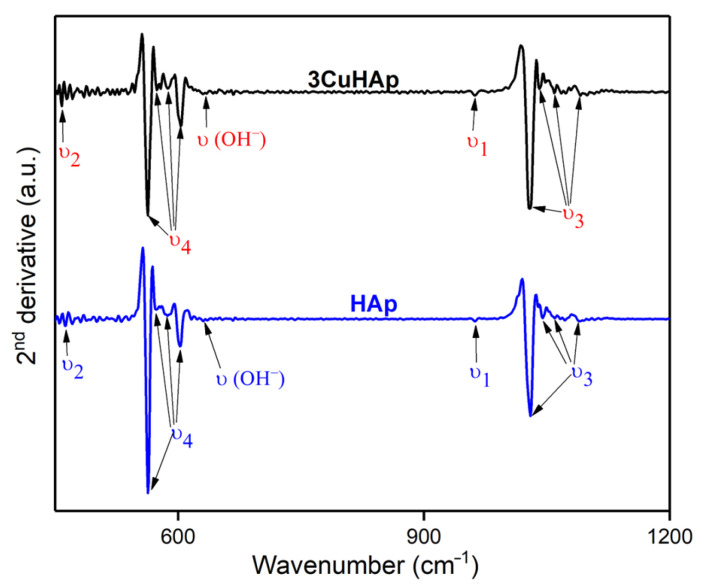
FTIR second-derivative spectra of the HAp and 3CuHAp coatings.

To acquire detailed information about the vibrational bands present in the HAp and 3CuHAp samples, we conducted an FTIR second-derivative analysis. The obtained second-derivative spectra (Figure 11), which pertain to the spectral regions characteristic of the ν_4_, ν_3_, ν_2_, and ν_1_ phosphate group vibrations, are illustrated in Figure 11. The ν_1_ vibration band of the phosphate group could be observed at around 962 cm^−1^ in the second-derivative spectra [16,27,49,50,51,52,53,54,55]. Another vibrational band observed in the Figure 11 belongs to the ν_4_ (observed at around 563 cm^−1^, 574 cm^−1^, 587 cm^−1^ and 605 cm^−1^) and ν_3_ (noticed at about 1030 cm^−1^, 1041 cm^−1^, 1059 cm^−1^ and 1096 cm^−1^) vibrations of the phosphate group [16,27,49,50,51,52,53,54,55]. Furthermore, the presence of the vibrational peak associated with the vibration of the hydroxyl group (librational mode) could be noticed in the spectra. Also, the band specific to the ν_2_ vibration could be observed in the spectra at around 460 cm^−1^ [16,27,49,50,51,52,53,54,55]. Table 3 summarizes the position of the vibrational bands observed in the second-derivative spectra obtained for the HAp and 3CuHAp coatings that are similar to the one observed in the FTIR spectra.

As can be seen in both second-derivative spectra, no intense vibrational bands were observed that could be associated with the presence of a supplementary phase or impurities. These results are consistent with those obtained by the XRD studies.

**Figure 12:**
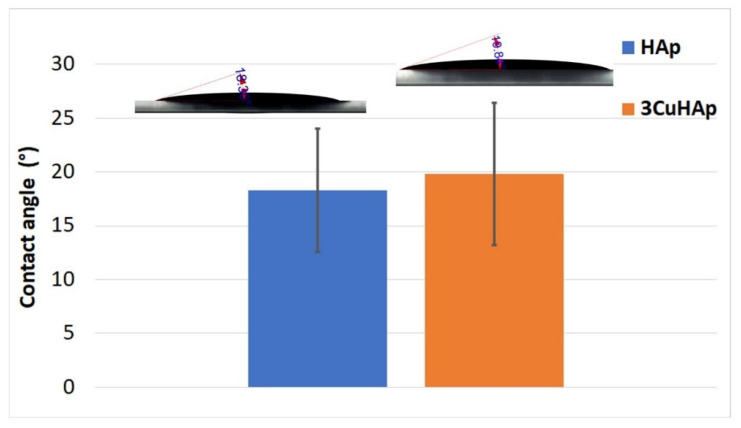
Water contact angle of the HAp and 3CuHAp coatings.

Wettability is a key property of implant surfaces that affects the cell–material interactions. Research has shown that hydrophilic surfaces promote enhanced mineral deposition and osteoblast cell growth in comparison to hydrophobic surfaces [52,56]. Therefore, in order to obtain valuable information about the wettability properties of the HAp and 3CuHAp coatings, water contact angle studies were performed (Figure 12). The mean value of the contact angle obtained for HAp was equal to 18.30 ± 5.7°. Meanwhile, the mean value obtained for the contact angle of the 3CuHAp coatings was 19.84 ± 6.6°. These values indicate the presence of a surface with a hydrophilic nature. As can be seen in Figure 12, by doping the hydroxyapatite with copper, a slight increase in the contact angle occurs. This behavior is consistent with the Wenzel’s relation for hydrophilic surfaces, which states that increasing the surface roughness will decrease the water contact angle value [57,58]. Such hydrophilic surfaces are known to enhance the biological properties of the materials, including the bioactivity and bone-bonding behavior [52,56]. Moreover, these results suggest that copper-doped hydroxyapatite coatings, when deposited through the vacuum deposition method, hold significant potential for improving the performance and safety of biomedical implants.

**Figure 13:**
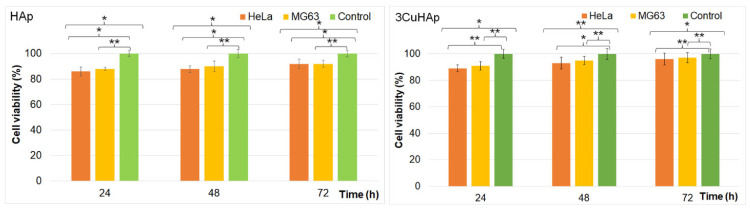
Graphical representation of the cell viability of HeLa and MG63 cells exposed to the HAp and 3CuHAp coatings for 24, 48 and 72 h. The results are depicted as the mean ± standard deviation (SD) and quantified as percentages of the control (100% viability). The ANOVA single-factor test was used for the statistical analysis and *p* ≤ 0.05 was accepted as statistically significant (* *p* < 0.03, ** *p* < 0.001).

The cytotoxicity of the HAp and 3CuHAp coatings was assessed using both the HeLa and MG63 cell lines by determining the cell viability with the aid of an MTT assay after 24, 48, and 72 h of incubation with the 3CuHAp coatings. The results of the MTT assays, as illustrated in Figure 13, are presented as the mean ± standard deviation (SD) from three experiments, with the cell viability expressed as a percentage of the control. The control was considered 100%. Statistical significance was determined using the ANOVA single-factor test, with *p* ≤ 0.05 considered to be significant. The data indicated that the viability of the HeLa and MG63 cells was not significantly different from the control after 24, 48, and 72 h of incubation with the HAp and 3CuHAp coatings, demonstrating good biocompatibility.

The viability of the HeLa and MG63 cells incubated with the HAp coatings was above 86% in the first 24 h and increased with the exposure time, reaching 92% after 72 h of exposure.

In the case of the 3CuHAp coatings, the cell viability remained above 88% after the initial 24 h and increased to 93% after 48 h and 95% after 72 h in the case of the HeLa cells. The result regarding the cell viability obtained for the MG63 cells was above 90% in the first 24 h and increased to 94% after 48 h and 96% after 72 h. These results are consistent with other reported studies regarding the biocompatibility of hydroxyapatite and hydroxyapatite composite coatings [16,18,59,60,61,62,63,64,65]. Furthermore, the cell viability of the HeLa and MG63 cells after exposure to the 3CuHAp coatings was consistently above 88%, exceeding the 70% cytotoxicity threshold set by ISO standard 10993-5 [59]. These findings suggest that the 3CuHAp coatings do not present a cytotoxic effect against HeLa and MG63 cells and could be promising candidates for the development of advanced implant materials with good biocompatibility.

**Figure 14:**
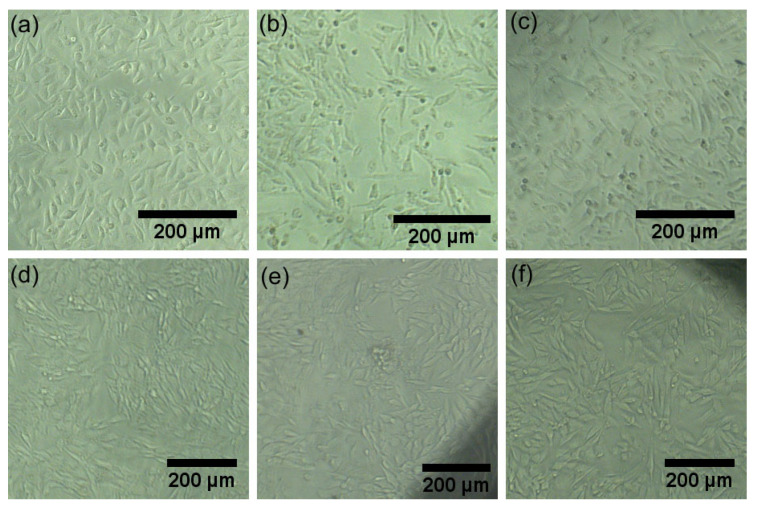
The morphology of HeLa and MG63 cells exposed to the HAp (**b**,**e**) and 3CuHAp (**c**,**f**) coatings for 72 h. HeLa control cells (**a**) and MG63 control cells (**d**).

Additional information regarding the biocompatibility of the HAp and 3CuHAp was achieved by visual observation of the morphology of the cells exposed to the HAp and 3CuHAp coatings for 72 h. The results revealed that the morphology of the HeLa and MG63 cells remained unchanged when cultured in the presence of both the HAp and 3CuHAp coatings (Figure 14). Moreover, the results also emphasized that the HeLa and MG63 cells seeded on the HAp and 3CuHAp coatings were allowed to proliferate for 72 h without altering their morphology. This observation supports the notion that the HAp and 3CuHAp coatings are biocompatible and could facilitate cell proliferation and tissue regeneration. Moreover, the results revealed that the integration of the implant into the surrounding tissue is possible. These biocompatible properties are attributed to both HAp, which is renowned for its osteoconductive nature and ability to mimic the mineral properties of bones, as well as the presence of the copper ions that form the HAp matrix, which plays an important role in bone growth [1,10,16,18,60,61,62,63,64,65,66,67].

**Figure 15:**
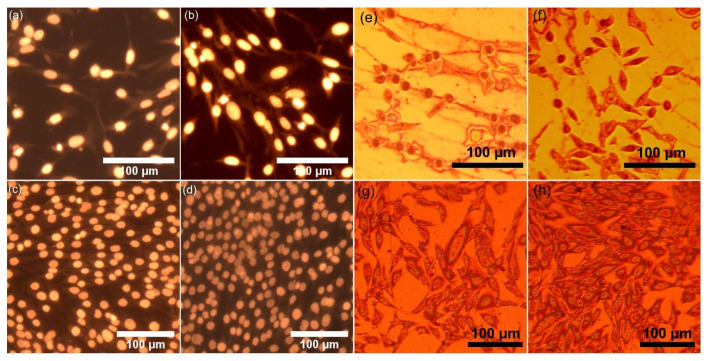
The morphology of HeLa and MG63 cells grown on the HAp coatings (**a**,**c**) and 3CuHAp coatings (**b**,**d**) visualized by fluorescence microscopy evaluation and the morphology of HeLa and MG63 cells grown on the HAp coatings (**e**,**g**) and 3CuHAp coatings (**f**,**h**) visualized by metallographic microscopy.

Additional information regarding the adhesion and proliferation of the HeLa and MG63 cells on the surface of the HAp and 3CuHAp coatings was obtained by fluorescence microscopy evaluation. For this purpose, the cells were allowed to grow on the surface of the HAp and 3CuHAp coatings and stained before observation. The images from the fluorescence microscopy studies are shown in Figure 15a–d. Furthermore, metallographic microscopy was used to assess the adherence and proliferation of the cells on the HAp and 3CuHAp coatings. The results of the metallographic microscopy are presented in Figure 15e–h.

The results of the visual observation revealed that the surfaces of both the HAp and 3CuHAp coatings allowed the studied cells to adhere and to develop. Furthermore, no morphological abnormalities were detected in the HeLa and MG63 cells that adhered to the surface of the coatings. Our studies reveal that the HeLa and MG63 cell viability increases with the time of exposure (from 24 h to 72 h) to the HAp and 3CuHAp coatings. The cell viability increases over time in the presence of the HAp and 3CuHAp coatings due to their biocompatibility, bioactivity, and ability to support cell adhesion, proliferation, and differentiation. Even though there are several specific mechanisms that are involved in cell adhesion and proliferation, depending on the cell type and the conditions of the experiment, the overall positive interaction between the cells and the HAp and 3CuHAp coatings is a consistent theme. Hydroxyapatite closely resembles the mineral component of bone, making HAp coatings well tolerated by various cells and tissues. They are bioactive, promoting cell adhesion, proliferation, and differentiation, and serve as a source of essential calcium and phosphate ions, enhancing cellular processes. The surface properties of HAp also support cell adhesion and protein adsorption, further enhancing the cell viability. HAp is also known to promote osteogenic differentiation in certain cell types, leading to increased cell function. Additionally, the low toxicity and ability to stabilize cell membranes reduces cell death and improves overall cell health. On the other hand, copper ions could increase the cellular viability by acting as essential cofactors for enzymes involved in various essential biological processes, such as cellular respiration, antioxidant defense, and collagen synthesis. Copper ions have the ability to protect cells from oxidative stress, support ATP production via the mitochondrial electron transport chain, and facilitate collagen and elastin cross-linking, which is crucial for tissue integrity. Furthermore, copper is also reported to be responsible for promoting angiogenesis, enhancing nutrient and oxygen supply. More than that, copper ions support immune function and regulate both the gene expression and signal transduction pathways, all of which collectively enhances cell growth, differentiation, and survival [10,11,12,13,68]. In this context, the increase in the HeLa and MG63 cell viability with the incubation time in the presence of the HAp and 3CuHAp coatings could be attributed to several factors: enhanced cell adhesion, improved nutrient exchange, and increased bioactivity, protection against cytotoxic agents, gradual ion release, and reduced oxidative stress. Together, these factors help to create a supportive environment for cells’ growth and survival.

Furthermore, to obtain a more comprehensive image of the biological properties of the 3CuHAp coatings, their antibacterial activity was also assessed against *Pseudomonas aeruginosa*, a common Gram-negative bacterial strain that is responsible for infections that are tough to treat in the blood, lungs, or other parts of the body.

**Figure 16:**
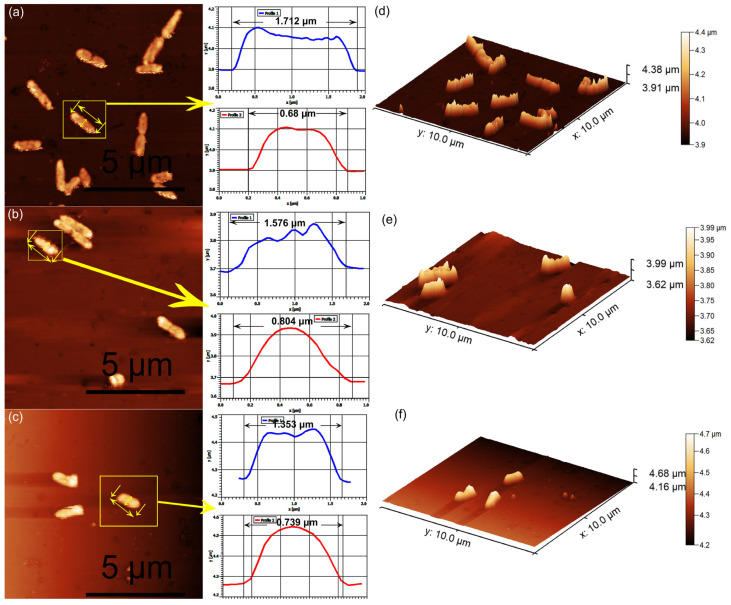
Two-dimensional AFM topography of *Pseudomonas aeruginosa* 27853 ATCC cells attached to the surface of the 3CuHAp coatings after a 24 (**a**), 48 (**b**) and 72 h (**c**) incubation period and their 3D representation (**d**–**f**). Individual bacterial cells chosen and their measured profile in width and in length, where the measurement is pointed out by the yellow arrow.

The ability of *Pseudomonas aeruginosa* cells to adhere to and develop on the surface of 3CuHAp coatings was investigated with the aid of AFM studies, with the aim of emphasizing the role of copper ions in combating bacterial growth. For this purpose, the AFM topographies of the 3CuHAp coatings were recorded after incubation with a *P. aeruginosa* bacterial suspension at various time intervals (24, 48, and 72 h), under ambient conditions and at room temperature. The 2D surface topographies of the 3CuHAp coatings exposed to the *P. aeruginosa* bacterial suspensions were captured in non-contact mode over an area of 10 × 10 µm^2^. The 2D AFM images of the 3CuHAp coatings, incubated with *P. aeruginosa* bacterial suspensions for 24, 48, and 72 h, along with their 3D representations, are displayed in Figure 16a–f.

The AFM recordings of the 2D surface topography of the 3CuHAp coatings demonstrated that these coatings effectively inhibited *P. aeruginosa* bacterial cell adherence and growth, even in the early stages of development. The AFM studies further highlighted the coatings’ ability to prevent *P. aeruginosa* biofilm formation on their surfaces. The adhered bacterial cells exhibited the characteristic rod-shaped morphology of *P. aeruginosa*, with lengths ranging from 1.03 to 2.57 µm and widths from 0.54 to 0.75 µm. The AFM data indicated that *P. aeruginosa* attachment and development on the 3CuHAp coating surfaces were significantly inhibited even within the first 24 h of incubation. Additionally, both the 2D topography and 3D representations suggested that the antibacterial activity of the coatings is correlated with the incubation time, showing a rapid decrease in the number of attached bacterial cells over time. After 72 h of exposure to the bacterial suspensions, the number of attached bacterial cells decreased significantly. The cell count performed on the AFM 2D images revealed that after 24 h of exposure, only 16 bacterial cells remained attached to the 3CuHAp surface, while after 48 h, only 9 bacterial cells were still adhered on the surface. After 72 h of incubation, both the 2D AFM topography as well as the 3D representation emphasized the presence of only a few isolated *P. aeruginosa* bacterial cells on the 3CuHAp coatings’ surface (the cell count revealed the presence of approximately three isolated bacterial cells). In addition, the AFM studies revealed that the 3CuHAp coatings inhibited *P. aeruginosa* biofilm formation.

**Figure 17:**
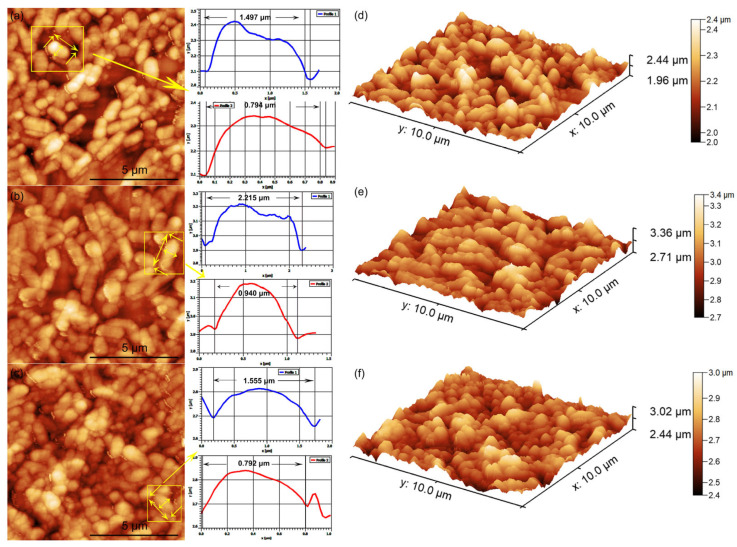
Two-dimensional AFM topography of *Pseudomonas aeruginosa* 27853 ATCC cells attached to the surface of the HAp coatings after a 24 (**a**), 48 (**b**) and 72 h (**c**) incubation period and their 3D representation (**d**–**f**). Individual bacterial cells chosen and their measured profile in width and in length, where the measurement is pointed out by the yellow arrow.

The antibacterial activity of the HAp coatings against *P. aeruginosa* was also evaluated. The results of the AFM topography recorded on the surface of the HAp coatings demonstrated that these coatings promoted the adhesion and development of *P. aeruginosa* on their surface, even from the early stage of the cell’s development. Furthermore, the data also showed that the HAp coatings provided the *P. aeruginosa* bacterial cells with an adhesive surface and allowed the development of bacterial biofilm. Also, the data suggested that the development of the bacterial cells on the surface of the HAp coatings increased with the incubation time. The data depicting the 2D topographies as well as the 3D representations of the HAp coatings’ surfaces after incubation with *P. aeruginosa* bacterial suspensions for 24, 48 and 72 are presented in Figure 17a–f. The results emphasized that since the HAp coatings did not exhibit any inhibitory activity against the *P. aeruginosa* bacterial cells, the antibacterial activity of the 3CuHAp could be attributed to the copper ions present from the HAp lattice. Furthermore, the data emphasized that the cells adhered to the entire HAp coating’s surface.

An individual analysis was also performed from the AFM data, which provided important quantitative measurements regarding the attached bacterial cell dimensions and surface roughness. The cross-section measurements along the lines depicted in Figure 16 and Figure 17, indicated by the yellow arrows, show information about the individual bacterial cell sizes. The results indicated that the adhered cells were well structured and integrated on the surface of the HAp coatings, and also that they were able to colonize the entire surface of the coatings. On the other hand, the results also emphasized that the bacterial cells were reduced in numbers and that the formation of biofilm was inhibited by the 3CuHAp coatings. From the profile images, the bacterial cells were observed to present the distinctive morphological features of *P. aeruginosa* bacterial cells, being relatively smooth and having lengths between 1.35 μm and 2.25 μm and diameters between 0.68 μm to 0.94 μm.

**Figure 18:**
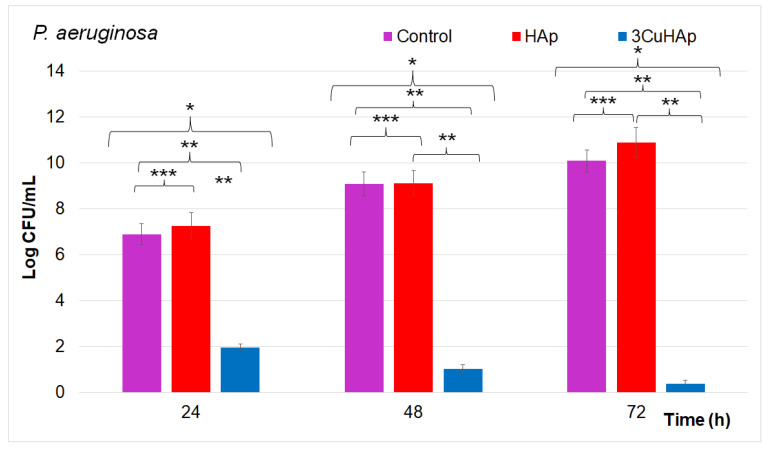
Graphical representation of the log colony forming units (CFUs)/mL of the HAp and 3CuHAp coatings incubated with *Pseudomonas aeruginosa* cells for 24, 48 and 72 h. The ANOVA single-factor test was used for the statistical analysis and *p* ≤ 0.05 was accepted as statistically significant (* *p* < 0.001, ** *p* < 0.002, and *** *p* < 0.007).

Additional information regarding the antibacterial activity of the HAp and 3CuHAp coatings was obtained by employing a quantitative in vitro antibacterial assay. The results of the quantitative studies are graphically represented in Figure 18. The quantitative assays depicted in Figure 18 highlighted the antibacterial activity of the 3CuHAp coatings on the adherence to and development of *P. aeruginosa* colony-forming units (CFUs) after exposure periods of 24, 48, and 72 h.

The antibacterial properties of the HAp and 3CuHAp coatings were examined, using a free *P. aeruginosa* culture as the positive control (C+). The results showed that the HAp coatings promoted the *P. aeruginosa* bacterial cell proliferation and positively influenced their development and adherence across all the tested time intervals. These findings are in good agreement with previous studies emphasizing that hydroxyapatite did not exhibit antibacterial activity [24]. The results of the quantitative antibacterial assays depicted in Figure 18 demonstrated that the development of *P. aeruginosa* CFUs was inhibited early within the first 24 h of exposure to the 3CuHAp coatings compared to the control. The in vitro antibacterial studies indicated a significant reduction in the *P. aeruginosa* colonies within the first 24 h compared to the positive control culture (C+) [69].

The results highlighted the crucial role of copper ions in the antibacterial properties of the 3CuHAp coatings, which were enhanced over time. The data indicated that the 3CuHAp coatings exhibited the strongest antibacterial effect after 72 h of incubation. Over this 72 h exposure period, the coatings, leveraging the antibacterial efficacy of copper ions, effectively inhibited the development of *P. aeruginosa* bacterial cells, significantly reducing the number of bacterial colonies. Even though the exact mechanisms responsible for the antibacterial behavior of the material are still unelucidated, there are several hypotheses. In this case, the most reported mechanism states that the exposure of bacteria to copper ions can interfere and modify the permeability of the cell membrane, leading to the leakage of cellular contents, and eventually, to cell lysis [70].

Another proposed mechanism affirms that copper ions could interfere with protein synthesis and with the DNA replication processes. The antimicrobial activity of copper ions has been attributed to their ability to generate reactive oxygen species (ROS) to inflict damage on bacteria [66]. Gram-negative bacteria have a negatively charged outer layer (lipopolysaccharides), which attracts cations and causes membrane damage. According to various studies [71,72,73,74], the anionic surface cell membrane promotes the binding of high concentrations of metal ions, enhancing toxicity. Copper ions can also interact with DNA by interfering with nucleic acid strands and disturb biochemical processes [75]. Copper ions can also generate intracellular hydroxyl radicals, which harm essential proteins [76].

The main mechanism of antimicrobial activity for metal ions is the generation of ROS, whether dependent or independent of Fenton reactions, and mainly leads to damage to cell membranes. Metals such as copper act as catalysts in Fenton-like reactions to generate ROS such as O2•, OH•, etc. [77], effects observed in the *E. coli* cell model [78]. Also, the free radicals created can cause damage to the mitochondrial membrane, resulting in cell degradation and ultimately cell death [71]. In addition, copper ions have been found to cause multiple toxic effects; in addition to generating reactive oxygen species, they can also lead to lipid peroxidation and protein oxidation [71], which ultimately lead to the stopping of cell division [79].

Even though the exact mechanisms responsible for the antimicrobial activity are still uncertain, it was hypothesized that the principal and most important of copper’s antibacterial mechanisms is attributed to the generation of reactive oxygen species (ROS), which induce oxidative stress that could damage bacterial cells. ROS damage various cellular components, including lipids, proteins, and DNA, leading to bacterial cell dysfunction and eventually death. Furthermore, copper ions have the ability to disrupt the bacterial cell membrane by binding and destabilizing their lipid bilayers, thus increasing the membrane permeability and causing leakage of its cellular contents. It was also reported that copper could interfere with the essential enzyme functions by binding to their active sites, further inhibiting bacterial growth and survival. This multifaceted strategy renders copper highly effective in preventing bacterial attachment, inhibiting biofilm formation, and ultimately, eliminating bacterial infections.

The data from the antibacterial assays are in good agreement with the present data from existing studies [66,67,80]. Furthermore, the results of the biological studies conducted emphasized that 3CuHAp possesses good biocompatibility and also exhibits strong antibacterial activity, making the coating potential candidates for future development in biomedical applications (coatings for various types of implants used in orthopedy and stomatology, medical devices, etc.).

The biocompatibility of the 3CuHAp coatings was thoroughly assessed using the HeLa and MG63 cell lines. The viability of the HeLa and MG63 cells following exposure to the 3CuHAp coatings was consistently above 88%, surpassing the 70% cytotoxicity threshold specified by ISO standard 10993-5. These results indicate that the 3CuHAp coatings exhibit no cytotoxic effects on HeLa and MG63 cells, thereby indicating their potential as promising candidates for the development of advanced implant materials with favorable biocompatibility. Furthermore, the microscopic observation showed that the morphology of the HeLa and MG63 cells remained unchanged when cultured in the presence of the HAp and 3CuHAp coatings. The antibacterial assays against *P. aeruginosa* also emphasized that the 3CuHAp exhibited significant antibacterial activity, even from the first 24 h of incubation. The findings of the AFM studies revealed that the 3CuHAp coatings effectively inhibited the formation of *P. aeruginosa* biofilms. Analysis of the AFM data highlighted a significant inhibition of *P. aeruginosa* attachment and development on the coating’s surface within the initial 24 h. Furthermore, both the 2D AFM topography and its 3D representation demonstrated the rapid decline of the attached bacterial cells over time, with a notable reduction observed after 72 h of exposure. The development of the 3CuHAp coatings represents a notable advancement in biomedical applications. The evident suppression of *Pseudomonas aeruginosa* biofilm formation and the notable decrease in bacterial cell attachment demonstrate the potential of these coatings to address infections linked with implantable medical devices.

In summary, our results suggest that the coatings based on HAp and 3CuHAp (Ca_10−x_Cu_x_(PO_4_)_6_(OH)_2_; x_Cu_ = 0, 0.03), as obtained by the vacuum deposition technique, exhibit structural, chemical, and morphological features that enhance their biological activity. The presence of copper induces good antibacterial activity to HAp. The coatings’ surface topography promotes enhanced in vitro biocompatibility. These findings underline that these types of coatings could represent promising candidates for uses in the biomedical field.

On the other hand, this study on copper-doped hydroxyapatite has some limitations, including a narrow range of doping concentrations, use of a single deposition technique and controlled laboratory conditions. Further research is needed and should explore a broader range of copper-doping levels, investigate alternative deposition methods, test long-term stability and analyze the mechanical properties, including in vitro and in vivo behaviors. These improvements will enhance the understanding and practical use of biomaterials based on copper-doped hydroxyapatite.

## 4. Conclusions

This study reported for the first time the development of copper-doped hydroxyapatite coatings by the vacuum deposition technique. Various techniques were used for the coatings’ complex characterization. In summary, the XRD studies confirmed the successful deposition of the HAp and 3CuHAp coatings, both containing pure hexagonal hydroxyapatite without impurities. The results of the AFM and SEM studies confirmed the continuous and homogeneous surface morphology of the coatings. Furthermore, the presence of hydroxyapatite in the studied coatings was proved by the FTIR results. The chemical composition of the HAp and 3CuHAp coatings was analyzed by XPS and EDS measurements. The results highlighted that the 3CuHAp coatings are highly biocompatible with the HeLa and MG63 cell lines, showing over 88% cell viability and no cytotoxic effects, making them suitable for advanced implant materials. Additionally, the in vitro antibacterial assays emphasized that the 3CuHAp coatings exhibit significant antibacterial activity against *Pseudomonas aeruginosa*, effectively inhibiting biofilm formation and bacterial attachment, highlighting their potential to prevent infections in implantable medical devices. Moreover, their proven compatibility with human cells indicates their potential integration into biomedical devices, thereby creating opportunities for the development of safer and more effective medical implants. In conclusion, copper-doped hydroxyapatite represents a promising candidate as a covering biomaterial for implants due to the superior biocompatibility and improved antimicrobial activity.

## Figures and Tables

**Table 1:** Roughness parameters of the HAp and 3CuHAp coatings’ surface obtained by AFM.

Sample	R_q_ (nm)	R_a_ (nm)
HAp	19.02	15.43
3CuHAp	18.73	15.16

**Table 2:** Roughness parameters of the HAp and 3CuHAp coatings’ surface obtained by SEM.

Sample	R_q_ (nm)	R_a_ (nm)
HAp	19.87	15.45
3CuHAp	18.79	14.78

**Table 3:** IR wavenumber positions (cm^−1^) specific to the ν_1_, ν_2_, ν_3_, and ν_4_ phosphate bands, as identified in the FTIR second-derivative spectra of the HAp and 3CuHAp coatings.

Assignments	Position (cm^−1^)	
	HAp	3CuHAp
Hydroxyl group	629	631
(ν_2_) phosphate groups	458	460
(ν_4_) phosphate groups	561, 573, 586, 602	563, 574, 587, 605;
(ν_1_) phosphate groups	960	962
(ν_3_) phosphate groups	1028, 1039, 1057, 1094	1030, 1041, 1059, 1096;

## Data Availability

The original contributions presented in the study are included in the article, further inquiries can be directed to the corresponding authors.
